# Regulation of *Pax6* by CTCF during Induction of Mouse ES Cell Differentiation

**DOI:** 10.1371/journal.pone.0020954

**Published:** 2011-06-13

**Authors:** Jie Gao, Jie Wang, Yumei Wang, Wei Dai, Luo Lu

**Affiliations:** 1 Division of Molecular Medicine, Department of Medicine, David Geffen School of Medicine, University of California Los Angeles, Torrance, California, United States of America; 2 Department of Environmental Medicine, New York University School of Medicine, Tuxedo, New York, United States of America; Florida State University, United States of America

## Abstract

*Pax6* plays an important role in embryonic cell (ES) differentiation during embryonic development. Expression of *Pax6* undergoes from a low level to high levels following ES cell differentiation to neural stem cells, and then fades away in most of the differentiated cell types. There is a limited knowledge concerning how *Pax6* is regulated in ES cell differentiation. We report that *Pax6* expression in mouse ES cells was controlled by CCCTC binding factor (CTCF) through a promoter repression mechanism. *Pax6* expression was significantly enhanced while CTCF activity was kept in the constant during ES cell differentiation to radial glial cells. Instead, the interaction of CTCF with *Pax6* gene was regulated by decreased CTCF occupancy in its binding motifs upstream from *Pax6* P0 promoter following the course of ES cell differentiation. Reduced occupancy of CTCF in the binding motif region upstream from the P0 promoter was due to increased DNA methylations in the CpG sites identified in the region. Furthermore, changes in DNA methylation levels *in vitro* and *in vivo* effectively altered methylation status of these identified CpG sites, which affected ability of CTCF to interact with the P0 promoter, resulting in increases in *Pax6* expression. We conclude that there is an epigenetic mechanism involving regulations of *Pax6* gene during ES cell differentiation to neural stem cells, which is through increases or decreases in methylation levels of *Pax6* gene to effectively alter the ability of CTCF in control of *Pax6* expression, respectively.

## Introduction

Regeneration of neural progenitor cells *in vitro* from embryonic stem (ES) cells is one of the promising methods to meet neurogenesis requirements in neurodegenerative therapy [1], [2], [3]. However, a major problem needs to be solved when inducing ES cells to generate neuronal stem cells is the heterogeneity. Improved methods have been developed to generate a certain type of neuronal stem cells, or called radial glial cells [4], [5], [6]. Radial glial cells have dual functions to produce neurons and to guide migration of the newly formed neurons [7], [8]. Recent studies demonstrate that treating ES cells with retinoid acid (RA) induces ES cell differentiation to become a pure population of the precursor cells that not only express a set of radial glial cell markers, but also have an enhanced expression of *Pax6*. However, *Pax6* rapidly fades away when radial glial cells start terminal differentiation becoming neurons [4], [5]. Due to dynamic changes in *Pax6* expression during committed neuronal differentiation of ES cells, *Pax6* may be a key factor that controls the specification of radial glial cell differentiation *in vitro*.

Homeobox transcription factor *Pax6* is highly conserved among vertebrate and invertebrate species and is crucial for the development of the eye, pancreatic islet cells and the central nervous system (CNS). *Pax6* mutations cause the small eye (Sey) defect in mice and ocular aniridia in humans [9]. During normal CNS development, *Pax6* regulates the balance between self-renewal and neurogenesis in neuronal precursors cells in a dose-dependant fashion, which reflects a need for a critical level of *Pax6* at the certain stage of neuronal differentiation [10]. However, there is a limited knowledge about regulation of *Pax6* in CNS development. Remaining questions are why there is a significant up-regulated *Pax6* expression in neural differentiation toward radial glial cells, and what causes down-regulation of *Pax6* during the terminal differentiation later. It is important to find answers for these questions in order to understand mechanisms involving regulation of *Pax6* and to ultimately control the process of neural differentiation.

In most species, *Pax6* transcription is regulated via P0 and P1 promoters [11], [12], [13]. There is a highly conserved transcription control element, termed ectoderm enhancer (EE). EE is located approximately −3.5 kbp upstream from the P0 promoter, which is important for promoting specific expression of *Pax6*, and to regulate retina- and neuro-specific gene expression [13]. Another regulatory element is located in intron 4 of the gene to direct activities of P0 and P1 promoters in the ciliary body, iris and amacrine cells [13]. The most recent report indicates that *Pax6* expression is regulated by CTCF, a zinc finger protein (ZFP) [14], [15], [16]. CTCF is a multivalent eukaryotic transcription factor that interacts with DNA sequences in the region of *Pax6* P0 promoter to block the interaction between the EE element and *Pax6* P0 promoter [15], [16], [17].

CTCF plays multifunctional roles in epigenetic regulation of DNA imprinting, X chromosome inactivation and transcriptional controls of gene expression [18], [19]. CTCF is initially characterized as a negative and positive regulator because of its capability to bind to DNA motifs in the promoter of various genes, including *c-myc*, p19ARF, p16INK4a, PIM-1, PLK, BRCA1, TERT and *Pax6* [16], [20], [21], [22], [23]. Later, there are new discoveries that demonstrate that CTCF functions as a unique insulator protein to regulate gene expression in both chicken globin and *h19/Igf2* loci [24], [25]. It also regulates communications between adjacent regulatory DNA elements in a position-dependent manner, or serves as a barrier to buffer transgenes from position effects caused by spread of the repressive heterochromatin from adjacent sequences [18], [23]. The binding of CTCF to insulator sequences or DNA boundary elements is often sensitive to modification of DNA methylation (CH_3_) that usually inhibits CTCF binding and eliminates CTCF-dependent actions [21], [26].

Previous study reveals that there is a repressor element located in promoter of *Pax6* gene containing five CCCTC motifs within a sequence of 80-bp nuclear acids. All of the five CCCTC motifs in this region are functional for the CTCF action [16]. The repressive effect of CTCF on *Pax6* expression is required for growth factor-induced proliferation [27]. In addition, ultraviolet (UV) irradiation inhibits CTCF expression that minimizes CTCF binding to the repressor element of *Pax6* [17], [28], [29]. In fact, over-expression of CTCF in transgenic mice suppresses *Pax6* gene expression resulting in retardation of embryonic ocular development including the cornea, lens and retina [15], [16]. In the present study, we generated neural stem cells from ES cells and obtained radial glial cells with enhanced *Pax6* expression. This *in vitro* differentiation procedure was applied as a working model to investigate the effect of CTCF on *Pax6* expression dynamically, and to further understand the regulatory mechanism underlying *Pax6* expression in early stages of ES and radial glial cells. Our data revealed an epigenetic mechanism indicating that increases and decreases in the methylation levels of *Pax6* gene effectively alter the ability of CTCF to control *Pax6* expression.

## Results

### Induction of radial glial cells from embryonic stem cells

**Figure 1:**
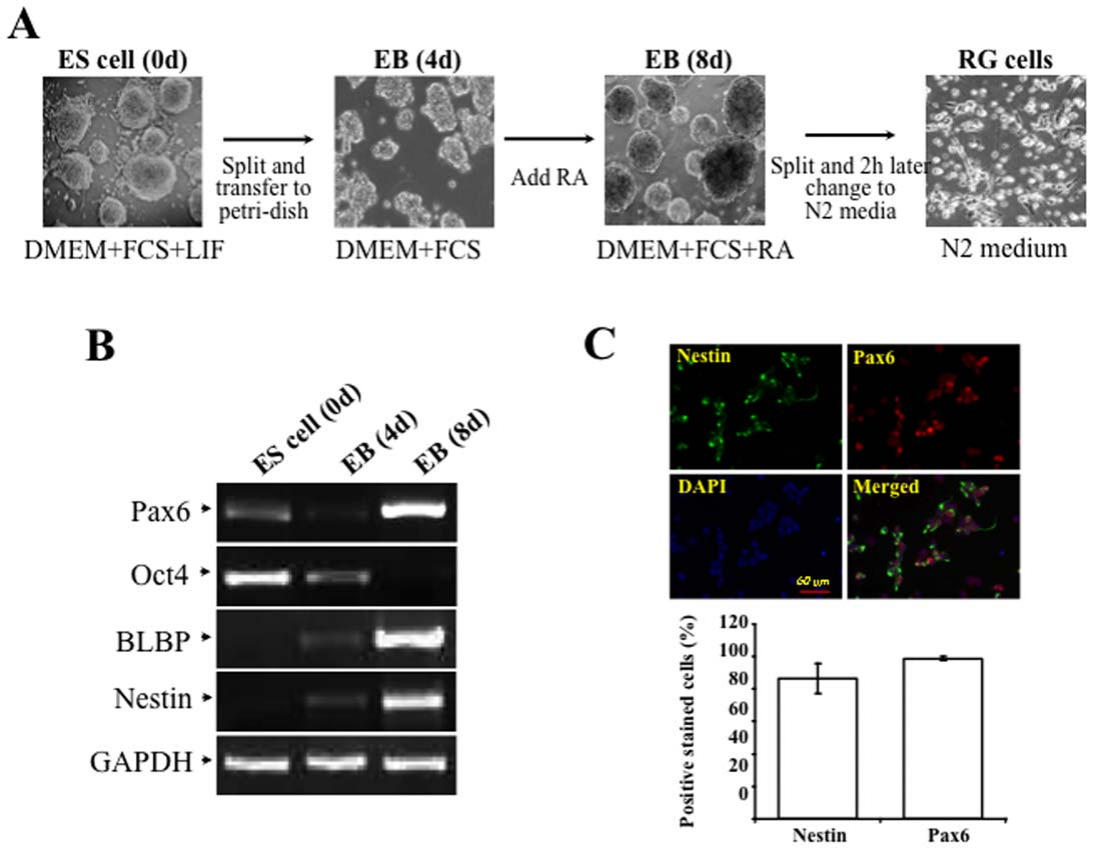
Induction of radial glial cells from embryonic stem cells. ***(A)*** Morphological changes of neural differentiation from ES cells. ***(B)*** Altered mRNA expression of ES cell markers (Oct4) and radial gial cells markers (nestin, BLBP and *Pax6*). ***(C)*** Staining for radial gial cells markers. ES cells growing on gelatin-coated plates were dissociated and transferred to nonadherent bacterial dishes to form embryonic body. After 4-day of embryonic body formation, 5 µM retinoid acid was added to the culture media and cells were allowed to grow for another 4 days. Embryonic bodies were dissociated after a total of 8-day induction and plated to poly-D-lysine/laminin-coated dishes in N2 medium. N2 medium is changed after 2 h. Cell samples were collected before and after the 4 days and days induction as shown as ES (0 d), EB (4 d) and EB (8 d) respectively. Photos of RG cells were taken 6 h after passage. Total RNAs were extracted at indicated time points and altered expressions of cell lineage-specific markers were monitored by RT-PCR. Cells were immunostained with antibodies specific to Nestin (green) and Pax6 (red). Cell nuclei were stained by DAPI (Blue). Results were plotted as a percentage of positively stained cells with Nestin and Pax6 antibodies vs numbers of DAPI-positive nuclei.

In the present study, a previous established protocol was used to induce ES cell differentiation toward radial glial cells [4], [5]. Cultured mouse ES cells were transferred to petri-dishes and treated with RA. Photos were taken by a Nikon invert microscope to document morphological changes of RA-induced ES cells in different stages of differentiation (*Fig. 1A*). Results of RA-induced ES differentiation followed a pattern that was consistent with previous reports. After formation of embryonic body (EB), RA-induced cells were successfully differentiated to spindle-shaped radial glial cells. Results from RT-PCR revealed that mRNAs of nestin (neural precursor marker) and BLBP (radial glial cell marker) were highly expressed, while expression of Oct4 mRNA (an ES cell marker) was suppressed in 8 days in RA-induced cells (*Fig. 1B*). More importantly, expression of *Pax6* mRNA was dramatically increased in the 8^th^ day in RA-induced cells. Nestin- and *Pax6*-positive cells were identified by immunostaining experiments in RA-induced cells, demonstrating that there was a significantly high conversion of ES cells to the radial glial cells (*Fig. 1C*). These results indicate that *Pax6* is regulated during ES cell differentiation toward radial glial cells.

### Regulation of *Pax6* expression by CTCF in ES cells

**Figure 2:**
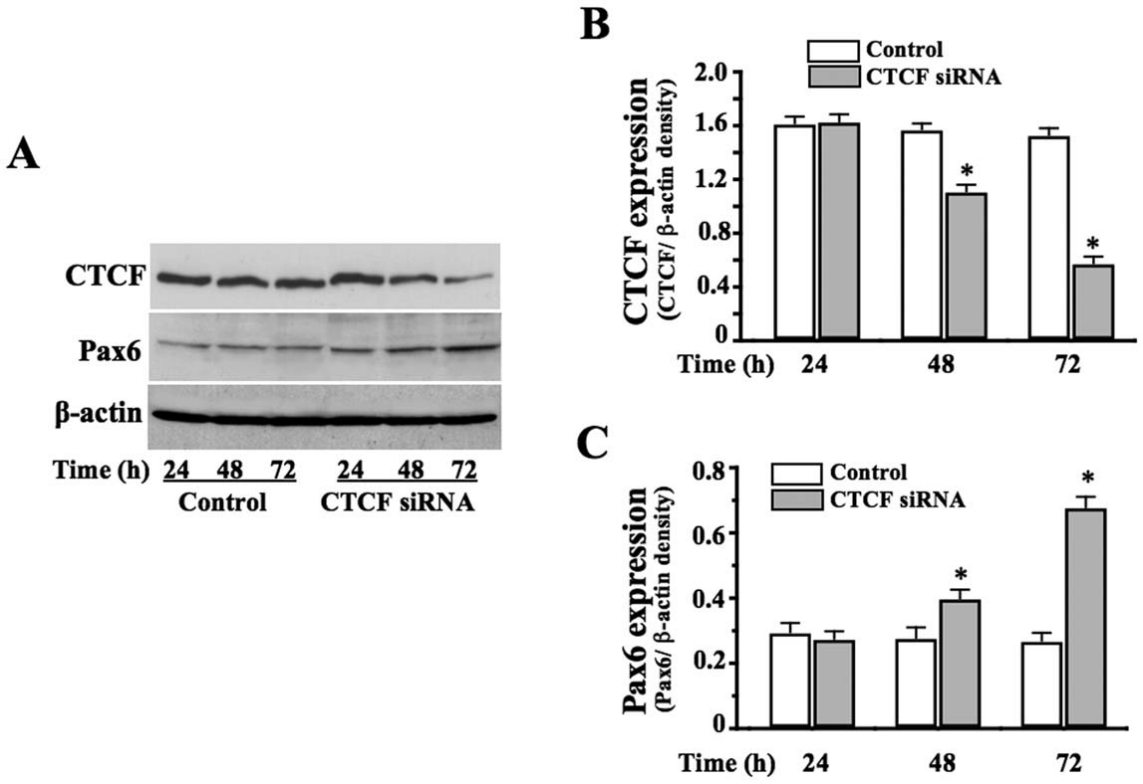
Effect of altered CTCF activity on *Pax6* expression in ES cells. ***(A)*** Effect of knocking down CTCF mRNA on *Pax6* expression. Statistical analysis of suppressed CTCF expression ***(B)*** and increased *Pax6* expression ***(C)***. Data was shown as mean ±S.E. Symbol “*” indicates significant differences (*p*<0.05, n = 4).

Our previous studies have demonstrated that CTCF is a negative regulator of *Pax6* gene in the cornea and retina. To study whether CTCF also involves regulation of *Pax6* in mouse ES cells, CTCF-specific siRNA was transfected to ES cells to knock down CTCF mRNA. We found that expression of CTCF at the protein level was decreased about 75% in ES cells transfected with CTCF-specific siRNA at 72 h (*Fig. 2A&2B*). At the same time (72 h), expression of *Pax6* protein was significantly increased following knockdown of CTCF (*Fig. 2A&2C*). For control experiments, ES cells were transfected with non-silencing control siRNA following the same procedure and time course as the experimental group. The results demonstrate that altered levels of CTCF by knockdown of CTCF mRNA in ES cells result in up-regulation of *Pax6* expression.

### Effect of RA-induced ES cell differentiation on CTCF activity

**Figure 3:**
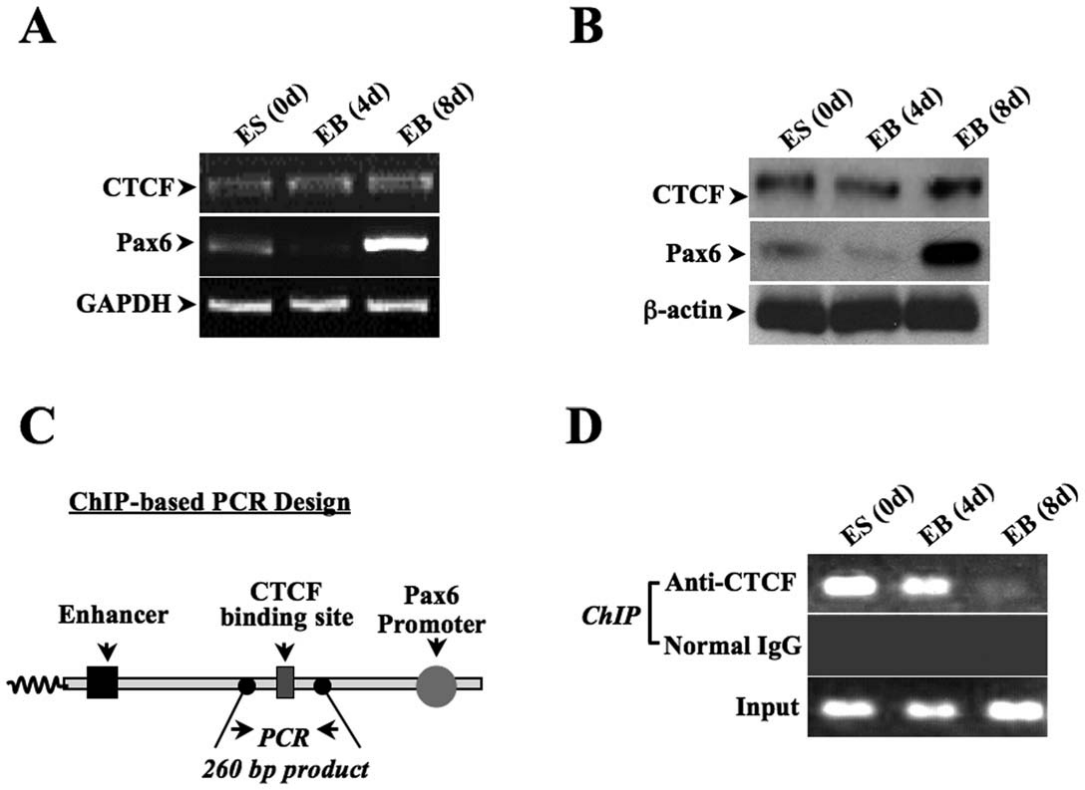
Correlation of CTCF and *Pax6* in ES cell differentiation to radial glial cells. ***(A)*** Expressions of CTCF and Pax6 mRNAs during ES cell differentiation to radial glial cells. Total RNA samples were harvested from undifferentiated ES cells (0 d) and embryonic body (EB) cells after 4 day or 8 day induction. RT-PCR assays were performed to measure the expression of CTCF, *Pax6* and GAPDH. ***(B)*** Expressions of CTCF and *Pax6* proteins during ES cell differentiation to radial glial cells. Western assays were performed to detect expression levels of CTCF, *Pax6* and β-actin. ***(C)*** Illustration of ChIP-based PCR designs. ***(D)*** Detection of specific protein-nucleotide interaction between CTCF and *Pax6* gene during ES cell differentiation and EB formation. Chromatin samples were immuno-pulled down by CTCF-specific antibody. ChIP-based PCR was performed to amplify CTCF binding DNA fragment in *Pax6* P0 promoter region. All ChIP and pCR experiments were repeated for at least three times.

Based on the observation that *Pax6* expression was regulated by CTCF in ES cells, we investigated whether CTCF activity was also changed to correlate to the altered *Pax6* expression during the time course of ES cell differentiation to radial glial cells. The effect of RA-induced ES differentiation on CTCF activity was investigated by focusing on two respects that include the CTCF expression level and its DNA binding capacity. First, both mRNA and protein expressions of CTCF were monitored following the time course of ES cell differentiation (*Fig. 3A&3B*). We found that *Pax6* expression markedly increased on the 8^th^ day of EB formation, and that there were no changes in CTCF expression at either the protein or mRNA level. Second, association of CTCF with the binding motifs in the promoter of *Pax6* gene was examined. It has been shown that CTCF binds as a repressor to the region of −1.2 kb upstream from *Pax6* P0 promoter to suppress *Pax6* transcription [16]. A set of primers was designed accordingly for chromatin immunoprecipitation (ChIP)-based PCR experiments to generate a 260 bp DNA fragment based on consensus DNA sequences upstream from the *Pax6* P0 promoter within the region of CTCF binding motifs (*Fig. 3C*). Results of ChIP-based PCR using specific antibodies against CTCF revealed that there were remarkable decreases in CTCF binding on DNA motifs in Pax6 promoter region on the 8^th^ day of EB formation. For controls, non-immunoprecipitated chromatins were used as DNA templates in PCR experiments (labeled as input) (*Fig. 3D*). Taken together, CTCF expression was not affected by ES cell differentiation, instead its binding ability to DNA motifs in Pax6 promoter was diminished, suggesting that increases in *Pax6* expression during differentiation to radial glial cells are very likely resulted from disengaging CTCF from its binding motifs and releasing *Pax6* promoter from the repressor effect of CTCF.

### Methylation-modification within CTCF binding region

**Figure 4:**
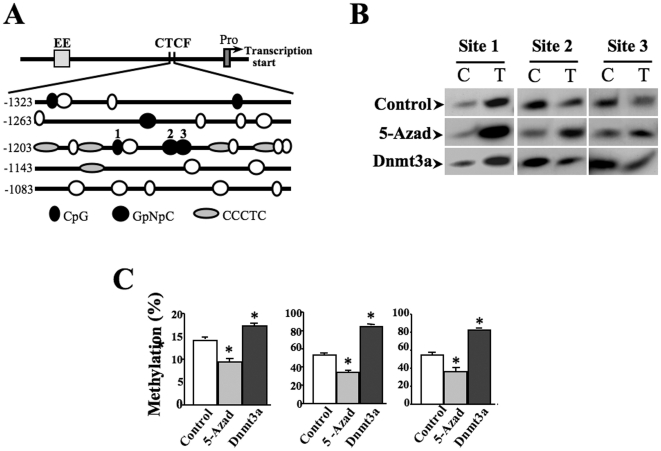
Identification of methylation sites within CTCF binding motifs. ***(A)*** Putative methylation sites in the region of CTCF binding motifs upstream from *Pax6* promoter. ***(B)*** Quantitative determination of methylation status at cytosines in CTCF binding sties after inductions of demethylation and methylation. Genomic DNA is treated with sodium bisulfite followed by PCR of the target sequence to generate the template for Ms-SNuPE assays. Methylation statuses were evaluated by the radio of radial incorporation of ^[32]^P-dCTP (representing methylated cytosine) and ^[32]^P-dTTP (representing unmethylated cytosines). DNA methylations were detected by using bisulfite sodium modifications and PCR. Demethylation and methylation were induced by 5-azadCyd (1 µM) and by over-expression of Dnmt3a, respectively. ***(C)*** Statistical analysis of methylation percentages in site1–3. Data was shown as mean ±S.E. and represented results from three independent Ms-SNuPE assays. Symbol “*” indicates significant differences comparing with cells without treatment. (*p*<0.05, n = 3).

It has shown that the interaction of CTCF with DNA sequence can be inhibited if the binding motif is methylated [18]. Next question is whether DNA methylation affects associations of CTCF with *Pax6* DNA since CTCF binding capacity to *Pax6* DNA decreased during radial glial cell differentiation. First, several potential 5-methylcytosines were located in the 80 bp region of CTCF binding motifs through DNA sequence analyzing (*Fig. 4A*). Genomic DNAs from ES cells were isolated and treated with sodium bisulfite that converts unmethylated cytosine to uracil while leaving 5-methylcytosine unchanged. DNA fragments containing CTCF binding sites were obtained by PCR with primers specific to bisulfite-converted DNA and sequenced for conformation. Normal genomic DNAs without sodium bisulfite treatment were also isolated for control experiments. In the PCR products, only 5-methylcytosines remained as cytosines (C) while un-methylcytosines were converted to thymines (T). Sequencing results revealed that within the tested DNA region, there were mixed C/T pairs occurring in 3 positions (sites 1–3 shown in *Fig. 4A*), indicating an uneven methylation of cytosine residues. All of other cytosine residues in CG-rich islands were completely converted to T (data not shown). Sites 1–3 were subjects to further quantitative analysis by performing Ms-SNuPE assays. These sites were differentially mehylated showing that methylations occurred about 15% in site 1, 55% in sties 2 and 3 (*Fig. 4B&4C*). To further verify methylation data, methylation responses of these sites were examined by altering cellular DNA methylation levels. ES cells were treated with 1 µM of 5-azadCyd to suppress methyltransferase resulting in decrease in DNA methylation. In contrast, ES cells were transfected with full-length cDNA encoding methyltranferase Dnmt3a to increase DNA methylation. Results showed: 1) site 1 was about 15% methylated in control cells compared to 12% and 18% methylated in 5-azadCyd treated and Dnmt3a-transfected cells, respectively; and 2) both sites 2 and 3 were approximate 55% methylated in control cells compared to 32% and 80% methylated in 5-azadCyd-treated and Dnmt3a-transfected cells, respectively (*Fig. 4C*). These results suggest that there are at least three cytosine residues that respond to alterations of cellular DNA methylation levels located in the region of CTCF binding motifs.

### Changed DNA methylation in CTCF binding region during differentiation

**Figure 5:**
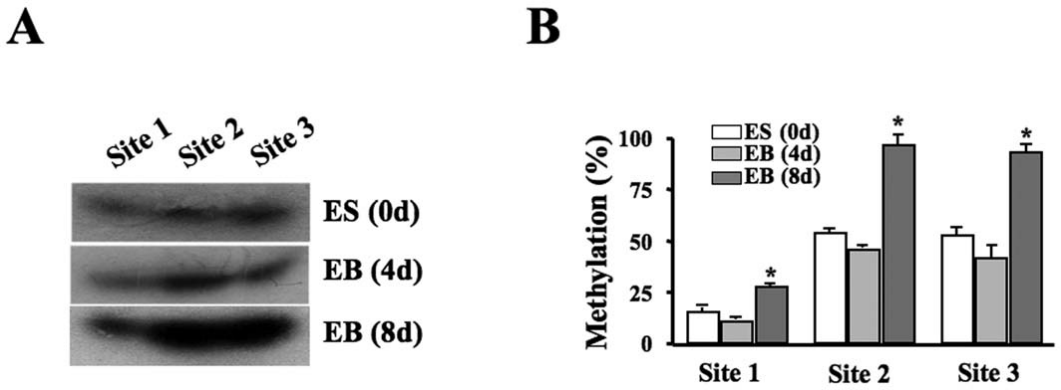
Methylation status of CTCF binding region in *Pax6* promoter during ES cell differentiation to radial glial cells. ***(A)*** Detecting changes of DNA methylation in CTCF binding sites during ES cell differentiation and EB formation. ***(B)*** Statistical analysis of DNA methylation changes in CTCF binding motifs during ES cell differentiation and EB formation. The genomic DNA harvested from ES cells and EB cells at different stages was treated with sodium bisulfite followed by PCR of the target sequence to generate the template for Ms-SNuPE assays. Methylated cytosines at site 1–3 were revealed by ^[32]^P-dCTP incorporation. Symbol “*” indicates the statistical significance determined by Student's t test at *p*<0.05 (n = 3).

DNA methylation statuses of 3 cytosine residues within sites 1–3 were monitored during ES cell differentiation to radial glial cells. DNA samples were taken from cells in three stages: un-differentiated ES cells before the induction, on EB 4^th^ day and EB 8^th^ day after the induction. Ms-SNuPE assays were preformed to quantitatively evaluate methylation levels of sites 1–3. Levels of 5-methylcytosines in sites 1–3 were measured by using ^32^P-dCTP incorporation. There were no significant changes in methylation levels in those measured sites between ES cells and EB at 4^th^ day (*Fig. 5A*). However, methylation levels were significantly increased in all of the three sites on the 8^th^ day of EB formation (*Fig. 5A&5B*). The results demonstrate an increased DNA methylation levels within CTCF binding motifs located in *Pax6* promoter region. Increases in DNA methylation in this region following the time course of ES cell differentiation may result in inhibition of CTCF binding to the *Pax6* promoter.

### Effect of DNA methylation on CTCF protein-nucleotide interaction

**Figure 6:**
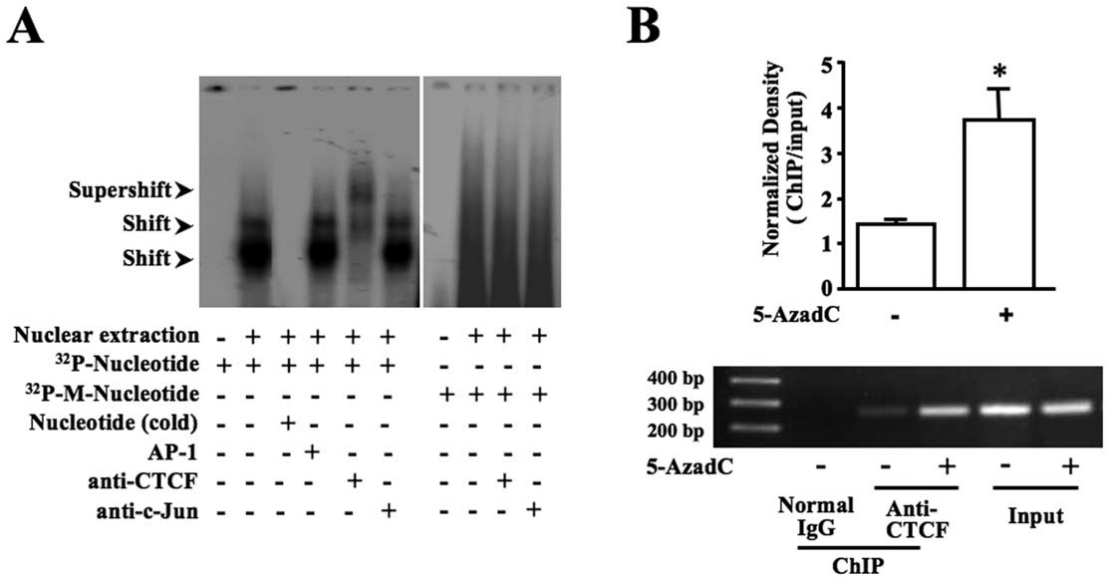
Effect of DNA methylation or de-methylation on protein-nucleotide interaction of CTCF and *Pax6* gene. ***(A)*** DNA and nuclear protein interactions were detected by EMSA using demethylated and methylated CTCF-specific DNA probes. Anti-CTCF antibody was used to detect DNA-protein complex super-shift. In addition, AP-1 DNA probe and *c-Jun* antibody were used in control experiments for DNA binding competition and super-shift experiments, respectively. Radioactive signals were visualized by exposure of the EMSA gel to X-ray film. ***(B)*** Significant increase in specific protein-nucleotide binding of CTCF to *Pax6* gene in demethylated ES cells. Chromatin samples in 1 µM 5-azadCyd-induced ES cells were immuno-pulled down by CTCF-specific antibody. ChIP-based PCR was performed to amplify the CTCF-binding motif DNA fragment in *Pax6* promoter region. Data were obtained from three independent ChIP and PCR experiments. Symbol “*” indicates significant differences (*p*<0.05, n = 3).

To study the specific effect of DNA methylation on CTCF binding capability to the *Pax6* promoter, EMSA/super-shift assays and ChIP-based PCR were performed to evaluate the DNA binding capability of CTCF. First, EMSA and super-shift assays were performed with synthesized and ^32^P-labelled DNA probes. These probes contained four CTCF binding sites in the 80 bp fragment upstream from the promoter. The probes were un-methylated or methylated in all of the three-cytosine sites. DNA-protein complexes were formed in nuclear extracts when those un-methylated probes were used and the binding between DNA and protein was competed by un-labeled probes, but not by nonspecific control probes (*Fig. 6A*). Furthermore, there was a super-shift band found in cell nuclear extracts incubated with CTCF-specific antibodies, however, there were no super-shift bands in the control sample treated with *c-Jun* antibodies. In contrast, there were no specific bands found in lanes that were treated with methylated probes, indicating that there was no DNA-protein complex formed (*Figure 6A*). The results demonstrate that DNA methylation negatively affects CTCF-specific binding activity in its motifs upstream from the *Pax6* promoter. To further verify the methylation-sensitive interaction between CTCF and the *Pax6* promoter, ChIP-based PCR were performed to detect the binding capacity of CTCF with the *Pax6* promoter in ES cells in the absence and presence of 5-azadCyd. Consistently, results of ChIP assays demonstrated remarkably enhanced bands in demethylation-induced ES cells when the CTCF specific antibody was used to pull down chromatins (*Fig. 6B*). Results from both EMSA and ChIP-based PCR experiments indicate that there are indeed methylation-sensitive interactions between CTCF and *Pax6* gene in ES cells.

### Effect of DNA methylation on *Pax6* expression

**Figure 7:**
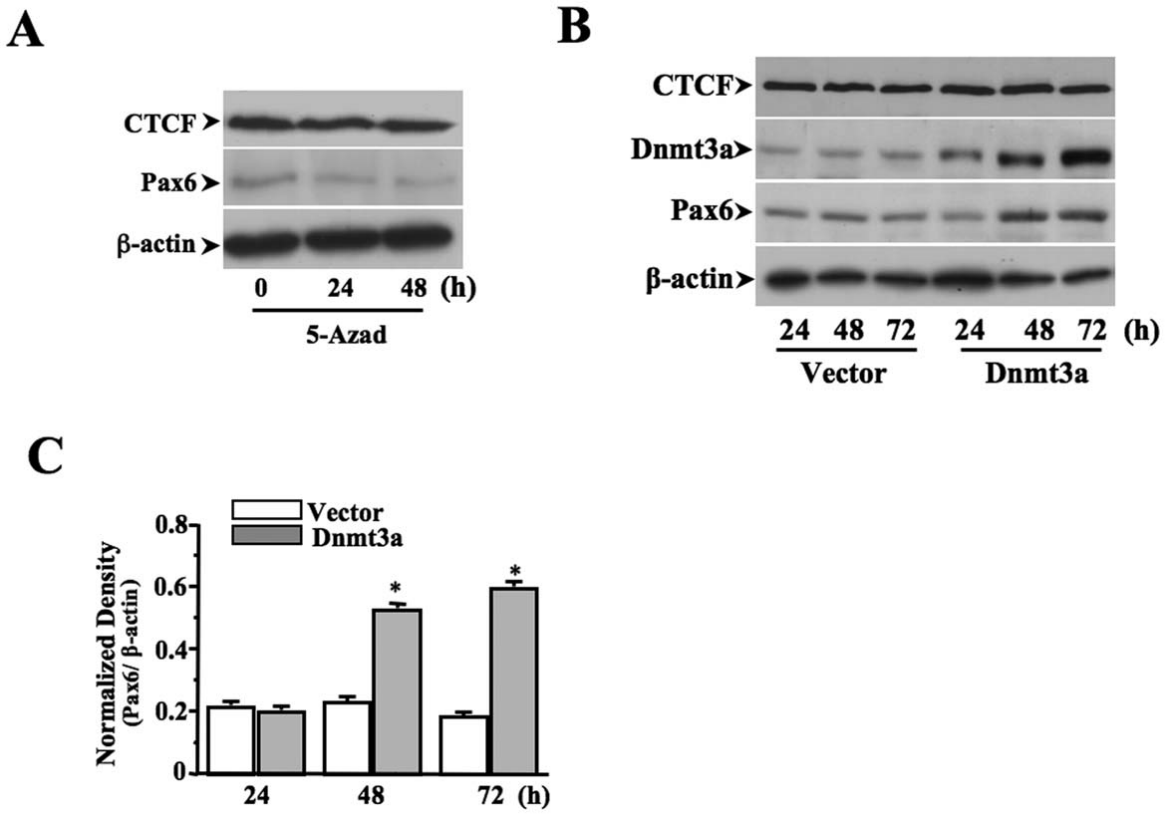
Effect of DNA methylation or de-methylation on *Pax6* gene expression. ***(A)*** Effect of decreasing DNA methylation on *Pax6* gene expression in ES cells. ES cells were treated with 1 µM 5-azadCyd for 24 and 48 h and subjected to Western analysis to compare the *Pax6* expression w/wo 5-azadCyd-mediated de-methylation treatment. ***(B)*** Effect of increasing DNA methylation on *Pax6* gene expression in ES cells. ES cells were transfected with full-length cDNA encoding Dmnt3a for 24–72 h and protein levels of *Pax6*, CTCF, Dmnt3a and β-actin were determined by Western analysis. (C) Data was shown as mean ±S.E. obtained from three independent experiments. Symbol “*” indicates significant differences between treated and un-treated groups.

Effects of altered DNA methylation on *Pax6* expression in 5-azadCyd-treated and Dmnt3a-transfected cells were monitored by Western analysis. Treatment of 5-azadCyd induced DNA de-methylation down-regulated *Pax6* expression (*Fig. 7A*). In contrast, over-expression of Dnmt3a by transfecting cells with full-length cDNA encoding Dnmt3 up-regulated *Pax6* expression following a time course (*Fig. 7B*). Statistical analysis showed that *Pax6* expression was significantly increased after over-expression of Dnmt3. The result is consistent with the observation that there was an increased DNA methylation in the CTCF binding motifs in the *Pax6* promoter region, and that there was an increased *Pax6* expression on the 8^th^ day of EB formation during ES cell differentiation to radial glial cells. Taken together, our data support the notion that there are increased DNA methylation in the region of CTCF binding motifs upstream from the *Pax6* promoter can exclude CTCF binding in the region during the period of ES cell differentiation to radial glial cells, which abolishes the repressor effect of CTCF on *Pax6* promoter activity, resulting in an enhanced *Pax6* expression.

## Discussion

Neural progenitor cells derived from ES cells can be a promising candidate for the cell-replacement therapy for neurodegenerative and retinal diseases [3]. To generate a homogenous resource of neural progenitor cells, it is important to identify key factors that can be used not only as an indicator of neural differentiation, but also as an entry point to manipulate the conversion course. *Pax6* appears to be a good candidate for this purpose since it is crucial for balancing neural stem cells self-renewal and neurogenesis [10], [30], [31]. There are three types of neural stem cells during CNS development, including neuroepithelial cells, radial glial cells and basal progenitor cells [7], [30]. *Pax6* is a key factor in the specification of radial glial cells [5], [32], [33]. Loss function of *Pax6* leads to the transition from predominantly radial glial cell-based neurogenesis to basal progenitor cell-based neurogenesis [30]. More importantly, regulation of CNS development by *Pax6* is sensitive to *Pax6* dosages that is evidenced by the followings: 1) heterozygous human mutation of *Pax6* results in aniridia and forebrain abnormalities; 2) homozygous human and mouse mutations of *Pax6* lack eyes and have marked microcephaly and absent olfactory bulbs; 3) increased expression of *Pax6* in transgenic mice results in microphthalmia and forebrain abnormalities [10]. In addition, during the course of *in vitro* conversion of ES cells to neural cells via an intermediate radial glial cell stage, *Pax6* expression exhibits extensive changes with the peak expression level in RG cells [4], [5]. In the present study, we have monitored *Pax6* expression patterns and the control of *Pax6* expression by CTCF in an established model *in vitro* during generating radial glial cells from ES cells. The mechanism involving CTCF occupancy and DNA methylation in CTCF-specific binding location in *Pax6* promoter explains how up-regulation of *Pax6* occurs in a certain stage of EB formation. All these *in vivo* and *in vitro* investigations are consistent with previous study results, indicating that *Pax6* expression must be under tight control during neural differentiation.

Differentiation of ES cells is initiated by cell aggregation in non-tissue culture plates, and then by adding retinoid acid on the 4^th^ day of EB formation. *Pax6* expression was tremendously enhanced on the 8^th^ day of EB formation accompanied with suppressed Oct4 expression, and with enhanced expressions of BLBP and nestin that are markers for radial glia and neuronal precursor cells (*Fig. 1*). These results are reminiscent of previous reports and prompted us to investigate what causes the dramatically increase in *Pax6* activity in radial glial cells [4], [5]. We consistently observed a slight down-regulated *Pax6* expression both at its mRNA and protein levels on the 4^th^ day of RA-induced differentiation. The association between CTCF protein and *Pax6* promoter may have not been affected yet on the 4^th^ day of the induction, suggesting that other mechanisms are involved in down-regulating *Pax6* expression. One possibility is that *Pax6* expression may be regulated by altered microRNA levels at this stage as it has been shown in neuron progenitor cells microRNA-9 indirectly inhibits Pax6 expression [34].

Previous studies found that the epigenetic regulator CTCF negatively controls transcription of the *Pax6* gene in ocular tissues [16]. Here, we demonstrate that CTCF also involved in regulation of *Pax6* in ES cells by showing that deficiency of CTCF by knocking down CTCF mRNA significantly could enhance *Pax6* expression (*Fig. 2*). Expression levels of CTCF mRNA and protein were not changed in the entire period of monitoring ES cell differentiation to radial glial cells. In stead, capability of CTCF interacting with its binding motif located upstream from *Pax6* promoter was remarkably reduced on the 8^th^ day of EB formation, providing a new explanation for increased *Pax6* expression at this stage that CTCF releases *Pax6* from a negative transcription control. The rest of the study contributed to investigate mechanisms underlying the decreased interaction of CTCF with its binding motif in *Pax6* promoter in radial glial cells.

Regulation of CTCF binding with its target DNA sequences is often through a methylation-sensitive mechanism. For example, CTCF epigenetically controls *IGF-2/H19* expression by binding to a differentially methylated domain (DMD) determined as imprinted control region (ICR) [26]. Three potential methylation sites (CG-rich islands) are tested inside the region where CTCF binding motifs are found. Site 1 was a low methylated site with approximately 15% methylation, suggesting that site 1 is unlikely to play a major role in methylation-modifed CTCF occupancy in control ES cells. However, methylation levels in sites 2 and 3 are rather higher in control cells and changed from 55% to 32% and 80% after 5-azadCyd-induced demethylation and Dnmt3a transfection-enhanced methylation, respectively (*Fig. 4*). The results not only demonstrate that there are 3 methylation sites inside of CTCF binding region, but also verify that 5-azadCyd treatment and Dnmt3a transfection can efficiently change methylation status of CTCF occupancy to interact with its binding motifs. Therefore, these results are helpful for us to understand the effect of DNA methylation on CTCF binding capability and control of *Pax6* expression. Methylation in all of the three sites has been quantitatively evaluated during ES cell differentiation. On the 8^th^ day of EB formation, hypermethylation occurred in sites 2 and 3 is remarkable, indicating these sites may play major roles in regulating CTCF occupancy and protein-DNA interaction with *Pax6* promoter. As reported in RA-induced ES cell differentiation to neuron progenitor cells, there are predominant gains of DNA methylation in promoters of several hundred genes. However, there are a little changes of globle DNA methylation between neuronal progenitors and terminal neurons [35]. This indicates that promoter hypermethylation is less dynamic during a transition to the terminal differentiation. We believed that there might be different mechanism for down-regulating Pax6 expression during terminal differentiation, such as polycomb-mediated repression. Polycomb targets are very dynamic during linage commitment and terminal differentiation [36], [37].

More evidence shows that DNA hypermethylation occurring within this region is parallel to decrease in CTCF occupancy and to increase in *Pax6* expression, suggesting that methylation of *Pax6* gene plays a functional role in regulating *Pax6* transcription that associates with ES cell differentiation. To establish the connection between releases of CTCF from its binding sites and hypermethylation in the same region, it requires more evidence to show whether mthylation/de-methylation regulates CTCF binding capability. To address this question, we performed EMSA and ChIP-based PCR to demonstrate the role that DNA methylation plays in the region of *Pax6* promoter to modulate the interaction between CTCF and its binding motifs (*Fig. 6*). It believes in general that methylation of CpG islands is a dynamic epigenetic marker that undergoes extensive changes during ES cells differentiation, which contributes to turn-on and turn-off of particular genes that are closely related to ES cell pluripotency and self-renewal [38], [39]. Thus, DNA methylation has become a target for developing pharmacological agents to manipulate directional differentiation of ES cells [40]. In the present study, hypermethylation in CTCF binding motifs in the *Pax6* promoter region occurring during ES cell differentiation functionally regulates *Pax6* activity associated with altering CTCF binding capacity. In summary, the present work provides a novel mechanism underlying the enhanced Pax6 expression during induction of neural progenitor cells from ES cells. The methylation-sensitive regulation of Pax6 during neural differentiation provides theoretical evidences for future development of techniques to better control neural progenitor cell generation from ES cells.

## Materials and Methods

### ES cell culture and treatments

Mouse J1 ES cells (purchased from ATCC) were cultured in Knockout™ D-MEM supplemented with 10% Knockout™ serum, 2 mM L-glutamine, 100 µM non-essential amino acid, 100 U/ml penicillin/100 µg/ml streptomycin and 1,000 U/ml ESGRO® in 0.1% gelatin coated dishes in a humidified incubator at 37°C with 5% CO_2_. All reagents for cell culture except for ESGRO® (Chemicon International Inc, CA) were purchased from Invitrogen™ Life Technologies, (Grand Island, NY). For ES differentiation experiments, ES cells were grown in non-adherent dishes in suspension to form embryoid bodies (EB). On the forth day of the EB formation, retinoic acid (5 µM) was added for additional 4-day period. EB cells were dissociated after the 8^th^ day, and cultured in poly-D-lysine/laminin-coated dished for 2 h in a complete DMEM medium supplemented with N2 supplement and human basic fibroblast growth factor (10 ng/ml). (Invitrogen, CA). Neuronal precursor cells were induced by further culturing EB cells in the neuronal precursors medium (Invitrogen, CA). The stock solution of 5-Aza-2′-deoxycytidine (5-AzadCyd, Sigma, St. Louis, MO) was made at a concentration of 1 mM. The final concentration of 5-AzadCyd used in treating ES cells was at 1 µM.

### Chromatin-immunoprecipitation (ChIP) and PCR

ES and EB cells were cross-linked in 1% formaldehyde for 10 min before the reaction was quenched by glycine at a final concentration of 125 mM for 5 min at room temperature. Cells were then homogenized in 1 ml cell lysis buffer to release nuclei. Isolated nuclei were re-suspended in 1 ml nuclear lysis buffer and genomic DNAs were broken down into 1∼2 kb fragments by sonication. Chromatin samples were immuno-precipitated by using CTCF-specific antibody (Millipore, Billerica, MA). The antibody-protein-DNA complexes were pulled down by protein A/G agarose pre-absorbed with salmon sperm DNA. Samples were then treated with proteinase K at 55°C overnight. Purified ChIP-DNA samples were analyzed by PCR using a pair of primers targeted to CTCF binding sites in the *Pax6* P0 promoter region (sense: 5′- TGTCGGGGGAGGAGCAAGAACC′ and antisense: 5′- CCTGGAGGGGCGGGAGACT-3′).

### Gene transfections and RNA interference

The plasmid pCMV-SPORT6 containing the cDNA fragment encoding full-length mouse cytosine-5-methyltransferase 3a (Dnmt3a) was purchased from ATCC® (MGC-5662). The pcDNA4 plasmid containing the cDNA fragment encoding full-length mouse CTCF was constructed in our lab previously [27]. Plasmids (20 µg per sample) were transfected into ES cells (1×10^6^ suspended in 600 µl of PBS solution) through electroporation at a setting of 250 V, 500 µF, and 75 Ω for 10 ms. Transfected cells were placed in full-growth medium and transferred to two 0.1% gelatin coated 60 mm culture dishes. Vectors without inserts were also transfected into ES cells for control experiments. For RNA interference experiments, specific primers that were complementary to CTCF mRNA (21 nucleotides plus other 8 nucleotides complementary to T7 promoter) were designed in our lab [27]. Briefly, a pair of primers was used with a sense strand sequence of “aaggaaugucuucuuuacacc”, and an antisense strand sequence of “aagguguaaagaagacauucc”. In addition, a nonsilencing control siRNA double-strand siRNA (sense: “aacauucgguagauuccucgc” and antisense: “aagcgaggaaucuaccgaaug”) was also synthesized and applied by using the same method. Sequence homologies of siRNA primers were examined by using a NIH Blast program. SiRNA was synthesized by *in vitro* transcription using a Silencer® siRNA Construction Kit (Ambion, Inc., TX). Double-strand RNA was synthesized by *in vitro* transcription using T7 RNA polymerase followed by RNase digestions to obtain siRNA. SiRNAs that were pre-mixed with Lipofectamine were used to transfect ES cells. None-specific siRNAs were synthesized using the same kit and transfected into ES cells for control experiments.

### Western analysis

ES cells were harvested by centrifugation at 2,000 g for 5 min. Cell pellets were resuspended in lysis buffer containing (in mM): 20 Tris-HCl-pH 7.5, 137 NaCl, 1.5 MgCl_2_, 2 EDTA, 10 sodium pyrophosphate, 25 β-glycerophosphate, 10% glycerol, 1% Triton X-100, 0.1% aprotinin, 0.1% leupeptin, 1 sodium orthovanadate, 1 phenylmethylsulfonyl fluoride. Equal amounts of denatured lysates (15 µg) were fractionated by SDS-PAGE and fractionated proteins in SDS-PAGE were electro-transferred onto a PVDF membrane. The membranes were incubated with primary antibodies in Tris-buffered saline containing 0.1% Tween 20 (TBST) containing 5% nonfat milk overnight at 4°C. All protein extracts were displayed in Coomassic blue-stained gels to ensure the equal amount of proteins being loaded. Antibodies against CTCF (sc-5916) and *Pax6* (sc-11357) were purchased from Santa Cruz Biotechnology. Antibody against Dnmt3a was purchased from Calbiochem (Cat. 317282). Mouse monoclonal antibody against β-actin (Sigma, St. Louis, MO) was diluted at a ratio of 1∶10,000. Specific signals were detected by using Western Blotting Luminol reagents (Santa Cruz Biotechnology, CA).

### Electrophoretic mobility shift assay (EMSA)

Two sets of double-stranded oligonucleotides (80 bp) with the specific sequence of *Pax6* promoter region contained all CTCF binding sites. Three of the binding sites were modified with/without replacements of 5-methylated cytosines (Operon Biotechnologies, Huntsville, AL). The probes were labeled with γ-^32^P-deoxy-ATP (∼3,000 mCi/mmol) using a T4 polynucleotide kinase labeling kit (Promega, WI) and purified with QIAquick nucleotide removal kit (Qiagen, CA). Nuclear protein extracts (10 µg) were incubated in the binding buffer containing 10 mM Tris-HCl (pH 7.5), 0.05 mg/ml poly (d*I*-d*C*), 0.5 mM EDTA, 50 mM NaCl, 1 mM MgCl_2_, 4% glycerol, and 0.5 mM DTT on ice for 15 min. Each binding reaction (20 µl) was initiated by adding 68.8 fmol probes and incubated on ice for 30 min. Gels were vacuum-dried and exposed to X-ray film for autoradiography. Competition experiments were performed by pre-incubating nuclear protein extracts with 1 pM of unlabeled probe on ice for 30 min and by adding different isotope-labeled probes. For super-shift assays, DNA-protein complexes were incubated for an additional 30 min using antibodies specific to CTCF and to negative control proteins.

### DNA preparation and bisulfite modifications

Cells were digested in lysis buffer (100 mM Tris-HCl [pH 8.5], 5 mM EDTA, 0.2% sodium dodecyl sulfate (SDS), 200 mM NaCl, 100 µg of proteinase K/ml) at 55°C followed by phenol-chloroform extraction. Total genomic DNAs were obtained by ethanol precipitated and cleaved by *EcoR I* digestion. The DNA fragments were modified by a bisulfite treatment following a method described previously [41], [42]. In brief, denatured DNA was mixed with freshly prepared 30 µl of 10 mM hydryquinone (Sigma, MO) and 520 µl of 3 M Sodium bisulfite (Sigma, MO) at pH 5.0. Samples were immerged in the mineral oil and incubated at 55°C for 6 h. Modified DNA was purified using the Wizard DNA Purification Resin kit (Promega, WI), and eluted into 50 µl of dd-H_2_O. Modification was completed by NaOH treatment for 10 min at 37°C and by ethanol precipitation. DNA was re-suspended in 20 µl of dd-H_2_O and used immediately or stored at −20°C.

### PCR amplification and DNA sequencing

Bisulfate-treated DNA (2 µl) was used as a template in strand-specific PCR amplification for the region of interest. Primer pairs were purchased from Operon Biotechnologies (Huntsville, AL). The PCR fragments were amplified under standard conditions with a total 35 cycles at different annealing temperatures followed by a final extension at 72°C for 10 min. Normal genomic DNA without demethylation treatment was used as a control. PCR product was purified and confirmed by DNA sequencing.

### Quantitative methylation analysis with Ms-SNuPE

Ms-SNuPE reactions were performed following the method described by Gonzalgo, et al. [43]. Briefly, purified PCR product (10 ng) was used in each reaction containing: 1× PCR buffer, 1 µM of each Ms-SNuPE primer, 1 µCi of ^32^P-labeled dCTP or dTTP and 1 U of Taq polymerase. Specific primers for Ms-SNuPE were: (site 1) 5′-TTTTTTTTTTTATTGTTTTTATT-3′, (site 2) 5′-TTTTATTT(C)GGTTAAGGAG TG-3′, and (site 3) 5′-TTTGGTTAA GGAGTGC(T)AGG. All samples were treated at 95°C for 1 min, 50–54°C for 2 min, 72°C for 1 min. Reaction products were loaded onto 15% denaturing polyacrylamide gels with 7 M urea. Results were visualized by exposure of X-ray films.

### Statistical Analysis

For Western and Chip analysis, signals in the films and agarose gels were scanned digitally and optical densities (OD) were quantified by using the Image Calculator software. The relative OD in Western analysis was calculated by normalizing the signals from target proteins against intensities of loading controls. In Chip assays, the relative density was calculated by normalizing PCR signals from immunopricipitated target chromatins against signals from the whole cell chromatin DNA. Data were plotted as Mean±SE. Significant differences between the control and treated groups were determined by One-way ANOVA and Student's *t* test at *P*<0.05.
